# Exosomal miRNA-215-5p Derived from Adipose-Derived Stem Cells Attenuates Epithelial–Mesenchymal Transition of Podocytes by Inhibiting *ZEB2*

**DOI:** 10.1155/2020/2685305

**Published:** 2020-02-21

**Authors:** Juan Jin, Yunguang Wang, Li Zhao, Wenli Zou, Mingming Tan, Qiang He

**Affiliations:** ^1^Department of Nephrology, Zhejiang Provincial People's Hospital, Zhejiang 310014, China; ^2^People's Hospital of Hangzhou Medical College, Zhejiang 310014, China; ^3^Institute of Nuclear-Agricultural Sciences, Zhejiang University, Hangzhou, 310058 Zhejiang, China; ^4^Quality Management Department, Zhejiang Provincial People's Hospital, Zhejiang 310014, China

## Abstract

**Background:**

Podocyte migration is actively involved in the process of podocyte loss and proteinuria production, which is closely associated with the development of diabetic nephropathy (DN). Exosomes from adipose-derived stem cells (ADSCs-Exos) effectively inhibit podocyte apoptosis in the treatment of DN. However, how ADSCs-Exos affect the migration of podocytes is obscure. This study is aimed at exploring the regulatory role of ADSCs-Exos on cell migration and the underlying mechanism.

**Methods:**

ADSCs-Exo was authenticated by transmission electron microscopy (TEM), western blotting, and flow cytometry. Cell viability and migration ability of podocytes were measured by CCK8 and Transwell assays, respectively. Relative expressions of miRNAs and mRNAs were determined by qRT-PCR. The transmitting between PKH26-labeled exosome and podocytes was evaluated by IF assay. Dual luciferase reporter assay was employed to detect the relationship between miR-215-5p and *ZEB2*.

**Results:**

The exposure to serum from DN patient (hDN-serum) significantly inhibited cell viability of podocytes, but ADSCs-Exo addition notably blunts cytotoxicity induced by the transient stimulus of hDN-serum. Besides, ADSCs-Exo administration powerfully impeded high glucose- (HG-) induced migration and injury of podocyte. With the podocyte dysfunction, several miRNAs presented a significant decline under the treatment of HG including miR-251-5p, miR-879-5p, miR-3066-5p, and miR-7a-5p, all of which were rescued by the addition of ADSCs-Exo. However, only miR-251-5p was a key determinant in the process of ADSCs-Exo-mediated protective role on podocyte damage. The miR-251-5p inhibitor counteracted the improvement from the ADSCs-Exo preparation on HG-induced proliferation inhibition and migration promotion. Additionally, miR-215-5p mimics alone remarkably reversed HG-induced EMT process of podocyte. Mechanistically, we confirmed that ADSCs-Exos mediated the shuttling of miR-215-5p to podocyte, thereby protecting against HG-induced metastasis, possibly through inhibiting the transcription of ZEB2.

**Conclusion:**

ADSCs-Exo has the protective effect on HG-evoked EMT progression of podocytes thru a mechanism involving ZEB2. Potentially, the ADSCs-Exo preparation is a useful therapeutic strategy for improving podocyte dysfunction and DN symptoms clinically.

## 1. Introduction

Diabetic nephropathy (DN) is the most common nephritic macrovascular complications of diabetes mellitus and an intractable kidney disease [1]. Glomerular filtration barrier (GFB) dysfunction is the typical clinical symptom of DN, which presents a microalbuminuria in the early stage of DN, the progression to evident proteinuria, and renal dysfunction over several years to decades, leading to end-stage renal disease (ESRD) [2–4]. Podocytes are one type of terminally differentiated visceral epithelial cells and an independent component of the GFB, which plays a vital role in keeping glomerular filtration barrier function [5]. Recently, researchers have found that hyperglycemia induces podocyte injury which is characterized by epithelial–mesenchymal transition (EMT) that is proposed as the causative factor of the GFB destruction and proteinuria development [6, 7]. However, the understanding of the pathogenesis of podocyte EMT is limited and effective strategies for reversing podocyte EMT are lacking.

Accumulating evidence has strongly revealed that exosomes derived from stem cells are safe and effective for treating renal diseases in rat and mouse models [8, 9]. Exosomes secreted by MSCs have a significant protective effect on renal injury in both acute kidney injury (AKI) and chronic kidney disease [8, 10]. Human urine stem cell-derived exosomes (USCs-Exo) reduce the apoptosis of podocytes and renal tubular epithelial cells and increase the proliferation of glomerular endothelial cells, leading to the decline of proteinuria excretion in DN rats [11]. Adipose-derived stem cell exosomes (ADSCs-Exos) effectively counter renal injury caused by acute ischemia injury [12] and attenuate chronic kidney disease (CKD) transition from acute kidney injury (AKI) by promoting tubular regeneration through activating Sox9 in renal tubular epithelial cells [13]. It has been identified that certain miRNA of stem cells is the main content which contributes to stem cell reprogramming and differentiation [14, 15]. Importantly, miRNAs can be packed in exosomes, acting as the potential paracrine regulator to participate in the modulation of many diseases such as ischemic diseases and degenerative ocular diseases [16–18]. Mesenchymal stem cell- (MSC-) produced microRNAs, such as miR-150 and miR-134, play a pivotal role in the treatment of DN [19]. Exosomes secreted by human urine-derived stem cells effectively alleviate the progression of DN and high glucose-induced podocyte injury by delivering microRNA-16-5p [20]. Our previous studies have demonstrated that ADSCs-Exos have a mitigative effect on DN disease by shuttling miR-486 to podocytes, thereby inhibiting Smad1/mTOR and activating autophagy flux [21]. In view of the positive relation between podocyte EMT and DN, and the fact that the autophagy inhibitor 3-methyladenine effectively reverses the effects of saponin astragaloside IV (AS-IV) on podocyte EMT [7, 22], we speculate that ADSCs-Exo may also attenuate DN by regulating the EMT progression of podocytes.

Herein, ADSCs-Exo with transcriptional functionally active miR-215-5p management successfully reduced high glucose-induced cell migration. ADSCs-Exo with low miR-215-5p presented a weaker protective role on HG-induced metastasis of podocyte compared to normal ADSCs-Exo. Therefore, the evidence is consistent with ADSCs-Exo having a protective effect in podocyte impairment through transmitting exosomal miR-215-5p.

## 2. Materials and Methods

### 2.1. Cell Isolation and Culture

ADSCs were harvested from 8-week-old C57BL/KsJ db/m male mice by utilizing the collagenase digestion method. Briefly, subcutaneous adipose tissue in the groin was isolated from anesthetized mice and washed with PBS buffer twice. Then, adipose tissues were minced and digested by collagenases for 40 min at 37°C followed by supplementing with a complete medium to stop enzymatic hydrolysis. The mixture was then filtered through a 40 mm cell strainer and centrifuged at 1500 rpm for 8 min. The sediment was resuspended and cultured in a RPMI-1640 complete medium at 37°C in a humidified incubator in 5% CO_2_. In the miR-215-5p inhibitor assay, ADSC cells were transfected with miRNA-215-5p inhibitor and negative control by Lipofectamine™ 2000 according to the instruction manual (11668-019, Invitrogen, USA) in ADSCs for 48 h. The miRNA inhibitors, including miR-215-5p, miR-879-5p, miRNA-217a-5p, miRNA-3066-5p, miRNA-7a-5p, and miR-215-5p mimics, were obtained from GenePharma, China.

Mouse MPC5 cells were cultured in a 5.5 mM D-glucose (NG) DMEM (10567022, Gibco, USA) containing with 10% fetal bovine serum and incubated at 37°C in a humidified atmosphere containing 5% CO_2_.

### 2.2. Exosome Isolation and Identification

Exosomes were collected from cell culture supernatant of ADSCs within passage 3 by using an exosome extraction kit (Wako Pure Chemical Industry, 293-77601) according to the manufacturer's specification. Briefly, ADSCs were firstly cultured in a 1640 complete medium allowing to grow to 70–80% confluence. Then, cells were incubated in a medium containing exosome-free FBS for another 24 h. Exosome-free FBS was obtained from fetal bovine serum (10099-141, Gibco, USA) which was ultracentrifuged at 100,000g for 18 h followed by filtration through a 0.22 *μ*m filter. After 24 h of incubation, the CM was collected and centrifuged at 300×g for 5 min, 1200×g for 20 min, and then 10,000×g for 30 min at 4°C to remove cells, debris, and large extracellular vesicles (EVs). Next, the CM were purified by an exosome extraction kit according to the manufacturer's specification. Then, exosomes were identified by transmission electron microscopy, western blotting, and flow cytometry used as described in previous reports and stored at −80°C for further studies [21]. The information of exosome markers was as follows: CD9 (bs-2489R, Bioss, China), CD63 (GTX41877, GeneTex, USA), and CD81 (GTX41794, GeneTex, USA).

### 2.3. CCK8 Assay

MPC5 cells were seeded in 96-well plates at a density of 3 × 10^3^ cells/well with a 5.5 mM D-glucose (NG) DMEM. After 12 h, MPC5 cells were exposed in a 5.5 mM D-glucose (NG) DMEM containing 10% serum from normal individuals (hH-serum) and DN patients (hDN-serum) followed by treatment with ADSCs-Exos at 0 h or 24 h. After 48 h, cells were added in 10 *μ*L CCK8 (P0010, Beyotime, China) reagents for 2 h and OD value was measured by a multiscan plate reader (m*μ*lISKANMK3, Thermo, USA) at the wavelength of 450 nm.

### 2.4. Transwell Assay

Briefly, MPC5 cells were seeded in 6-well plates with a density of 5 × 10^5^ cells/well. After transfection with certain miRNAs using Lipofectamine™ 2000 (11668-019, Invitrogen, USA), the cells were reseeded at a cell density of 2 × 10^5^ cells/well into the upper chamber of a Transwell apparatus in a 24-well plate (353097, BD Biosciences, USA) with a free-serum medium. The lower chamber was filled with a complete medium. The cells were incubated in a medium containing 5.5 mM D-glucose (NG), 5.5 mM D-glucose+24.5 mM mannitol (MA), or 30 mM D-glucose (HG) for 24 h cells followed by treatment with ADSCs-Exo for another 48 h. Then, cells on the upper surface of the membrane were cleaned and cells on the lower surface of the membrane were fixed with 70% cold-ethanol and stained with 0.1% crystal violet. Cell migration area was recorded by using an inverted microscope (IX51, OLYMPUS, Japan). The number of stained cells was counted under a microscope.

### 2.5. Real-Time Quantitative PCR (qRT-PCR)

Total RNA was extracted with Trizol (15596026, Invitrogen, USA) under the manufacturer's instructions, and 1 *μ*g RNA was reverse-transcribed into cDNA by using the cDNA reverse transcription kit (4368813, Applied Biosystems, USA). Real-time quantitative PCR (qRT-PCR) was performed to measure the relative mRNA expression of target genes by using SYBR Green (Q111-02, VAZYME, China). All data were normalized to the control of GAPDH or U6. PCR primer sequence is shown in Table 1. 2^*ΔΔ*Ct^ was used to calculate the relative expression of these genes.

### 2.6. Exosome Uptake Assay

ADSCs-Exos were labeled with the membrane fluorescent dye PKH26 (CD117, Sigma-Aldrich, USA) according to the manufacturer's instruction. Briefly, purified exosomes from ADSCs or ADSCs transfected with miR-215-5p were resuspended in 1 mL of diluent C and labeled with PKH26. Then, the exosomes were added to MPC5 cells at the same dose for 24 h incubation. Cells were fixed in 4% paraformaldehyde for 10 min. After rinsing with by PBS, cells were stained with DAPI and the PKH26-labeled exosomes in sensitive cells were observed by a laser confocal microscope (TCS SP5, Leica, Germany).

### 2.7. Dual-Luciferase Assay

The normal sequence of *ZEB2-*3′ UTR containing a miR-125-5P binding site and its mutated 3′ UTR sequence were synthesized by PCR amplification and integrated into the pYr-MirTarget basic vector. HEK293T cells were seeded in 24-well plates with a density of 5 × 10^4^ cells/well. After 12 h, cells were transfected with 50 nM miR-125-5p mimic or negative controls by using Lipofectamine™ 2000, followed by cotransfection with 2 *μ*g of the WT or 3′ UTR-mutant of ZEB2, respectively. Luciferase activities were performed under the instruction of Luciferase Reporter Detection Kits (C0037, Promega, USA) at 48 h posttransfection. Each sample was duplicated at least 3 times. The sequences were as follows: ZEB2-3′UTRMUT F:CATTTAATTTAACGACTAATAACATTTTATTTATGTGG, ZEB2-3′UTRMUT R:AAAATGTTATTAGTCGTTAAATTAAATGAATGCAAAAA.

### 2.8. Western Blotting

Total proteins were extracted by using RIPA protein lysate (89900, Invitrogen, USA). 30 *μ*g of total protein was separated by 10% SDS-PAGE. Primary antibodies against E-cadherin (1 : 1000, AF0131, Affinity Biosciences, USA), *α*-SMA (1 : 1000, XBT-1, Affinity Biosciences, USA), ZEB2 (1 : 1000, AF5278, Affinity Biosciences, USA), GAPDH (NCI5079, Good Here, China), CD9 (1 : 1000, bs-2489R, Bioss, China), CD63 (1 : 1000, GTX41877, GeneTex, USA), and CD81 (1 : 800, GTX41794, GeneTex, USA) were employed to incubate with activated membrane overnight at 4°C. Then, the bands were detected by incubation with HRP conjugate (1 : 5000, BA1054, Boster, China) secondary antibody at room temperature for 2 h. Chemiluminescence detection was conducted using an enhanced chemiluminescence reagent (P0018AS, Beyotime, China), and the images were observed by a gel imaging analysis system (UniCel DxI800, Beckman Coulter, USA).

### 2.9. Statistical Analyses

All experiments were repeated at least 3 times. Relative quantitative analysis of protein and cell counting was processed using ImageJ software. All values were presented as means ± SEM, and these data were statistically analyzed by a one-tailed Student's *t* test and one-way ANOVA by using GraphPad Prism 6.0 software. Statistically significant differences were accepted at *P* < 0.05.

## 3. Results

### 3.1. ADSCs-Exos Attenuate High Glucose-Induced Migration and Damage in MPC5 Cells

**Figure 1:**
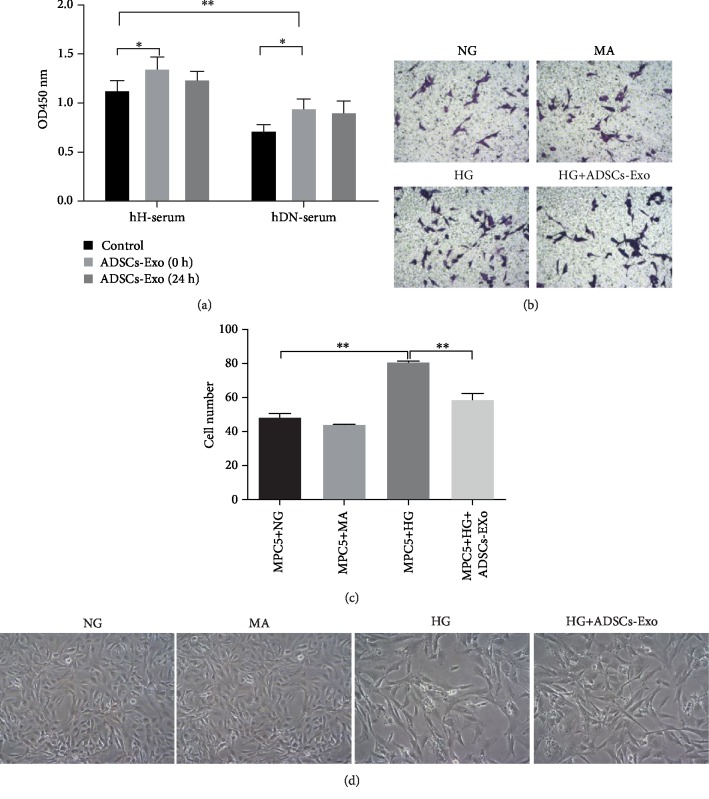
Exploration of the role of ADSCs-Exos on HG-induced migration of MPC5 cells. (a) MPC5 cells were incubated with sterile serum from control and DN patients supplemented with or without ADSCs-Exos, and then, cell viability was measured by CCK8 assay. (b) Representative images of migration cells as evaluated by Transwell assay in HG-induced MPC5 cells treated with NG, MA, HG, and ADSCs-Exos. (c) The number of migratory cells was counted by ImageJ software in HG-induced MPC5 cells treated with NG, MA, HG, and ADSCs-Exos. (d) Photograph of cellular damage in different groups was observed by a biological microscope. NG: normal glucose; HG: high glucose; MA: mannitol. ^∗^*P* < 0.05; ^∗∗^*P* < 0.01.

Our previous study demonstrated that ADSCs-Exos have protective action on DN by curbing apoptosis in podocyte [21]. Here, ADSCs-Exos were also successfully extracted from a culture medium of mouse adipose stem cells. As shown in [Supplementary-material supplementary-material-1], circular particles were vividly observed under transmission and electron microscopy (Fig. [Supplementary-material supplementary-material-1]). Exosome specific surface markers including CD9, CD63, and CD81 exhibited extremely high expression which were not expressed in ADSCs (Fig. [Supplementary-material supplementary-material-1]). Most specifically, 86.7% of CD9-, 94.5% of CD63-, and 90.8% of CD81-positive particles were detected in the isolated ADSCs-Exos by flow cytometry (Fig. [Supplementary-material supplementary-material-1]). Similarly, once treated with serum from DN patients (hDN-serum), the cell viability was significantly inhibited compared to cells exposed to serum from healthy individual (hH-serum). Treatment with ADSCs-Exos enhanced the cell viability notably, both in the hH-serum and in the hDN-serum-treated group. When MPC5 cells were firstly treated with serum from healthy and DN patients for 24 h, the additive treatment of ADSCs-Exo for the next 24 h did not induce the proliferation of podocyte significantly, implying a transient protection action of ADSCs-Exos on podocyte injury (Figure 1(a)). With the migration chamber assay, we further confirmed that high glucose significantly accelerated MPC5 cells' migration, while this effect was powerfully attenuated by ADSCs-Exo administration (Figures 1(b) and 1(c)). Also, ADSCs-Exos also effectively reversed HG-induced morphological damage and cell loss in MPC5 cells (Figure 1(d)). Together, these results indicate that ADSCs-Exos restore the HG-induced metastasis and impairment in podocytes.

### 3.2. The miR-215-5p Is Essential for the ADSCs-Exo-Mediated Improvement of Podocyte Migration

**Figure 2:**
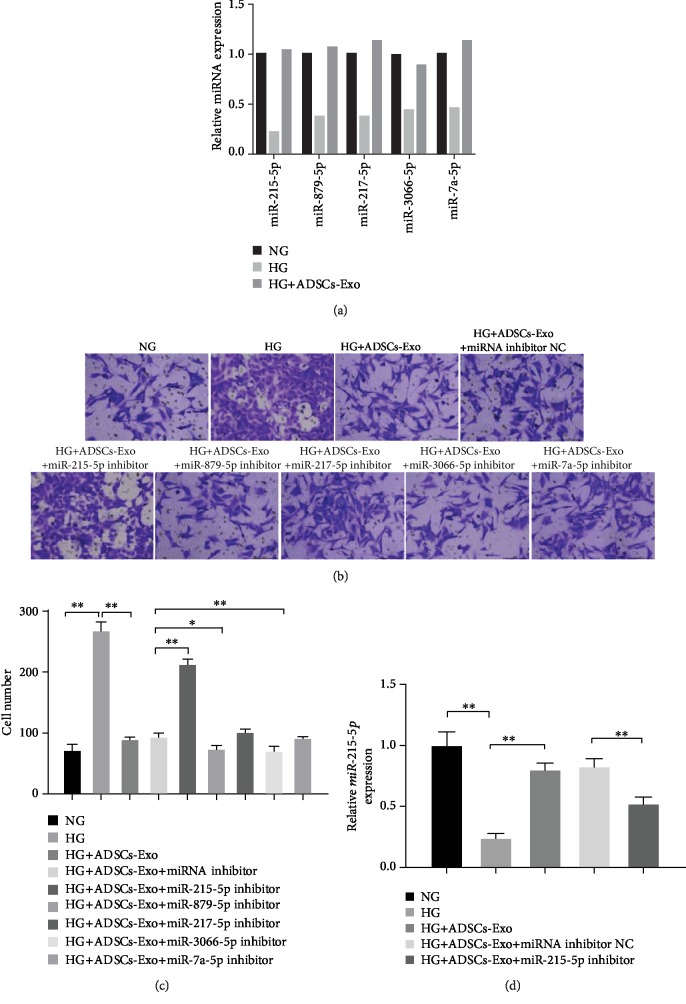
miR-215-5p mediated the protective effects of ADSCs-Exos on HG-induced migration of MPC5 cells. (a) The expression of EMT-related miRNAs including miR-215-5p, miR-879-5p, miRNA-217a-5p, miRNA-3066-5p, and miRNA-7a-5p was determined by qRT-PCR in HG-induced MPC5 cells with or without ADSCs-Exo treatment. (b) Cell migration was evaluated by Transwell assay in HG-induced MPC5 cells treated with ADSCs-Exo NC and ADSCs-Exo-miRNA inhibitors. (c) The number of migratory cells was counted by ImageJ software in different groups. (d) The expression of miR-215-5p was measured by qRT-PCR in HG-induced MPC5 cells treated with ADSCs-Exo NC and ADSCs-Exo-miR215-5p inhibitor. ^∗^*P* < 0.05; ^∗∗^*P* < 0.01.

To explore the underlying mechanism of ADSCs-Exo on podocyte injury, we evaluated the expression pattern of EMT-related miRNAs including miR-215-5p, miR-879-5p, miRNA-217a-5p, miRNA-3066-5p, and miRNA-7a-5p in cells treated with HG. Interestingly, the expression of these miRNAs was strongly decreased in the HG groups compared to the control groups, but addition of ADSCs-Exos significantly enhanced HG-induced miRNA expression (Figure 2(a)). Through the migration chamber assay, we observed that HG-induced podocyte migration was dramatically blocked by ADSCs-Exos, while the transfection of miR-215-5p inhibitor impaired the protection performance of ADSCs-Exos compared with the miR-215-5p negative control transfection group (Figure 2(b)). The number of migratory cell that verified miR-215-5p was required for ADSCs-Exo-mediated protection effect in HG-induced podocyte EMT, while miR-879-5p and miRNA-3066-5p inhibitor addition further augmented the improvement role of ADSCs-Exo on cell migration. Unfortunately, the data showed no significant effects of miRNA-217a-5 and miRNA-7a-5p inhibitors on this process (Figure 2(c)). The inhibition efficiency of miR-215-5p, miR-879-5p, miRNA-217a-5p, miRNA-3066-5p, and miRNA-7a-5p inhibitors was identified and showed a powerful inhibitory effect (Fig. [Supplementary-material supplementary-material-1]). Given the key role of miR-215-5p, we evaluated the relative expression of miR-215-5p in MPC5 cells treated with HG, ADSCs-Exos, and miR-215-5p inhibitor. As shown in Figure 2(d), the relative expression of miR-215-5p was more robustly declined in the HG groups than in the control groups, which was effectively recovered under the condition of ADSCs-Exo. However, the miR-215-5p inhibitor obviously suppressed the upregulation of miR-215-5p induced by ADSCs-Exos (Figure 2(d)). These data imply that ADSCs-Exo management possibly mediate the shuttling of miR-215-5p to recipient cells, thereby leading to the expression of miR-215-5p and metastasis of MPC5 cells.

### 3.3. The miRNA-215-5p Prominently Suppressed HG-Induced MPC5 Cell Migration

**Figure 3:**
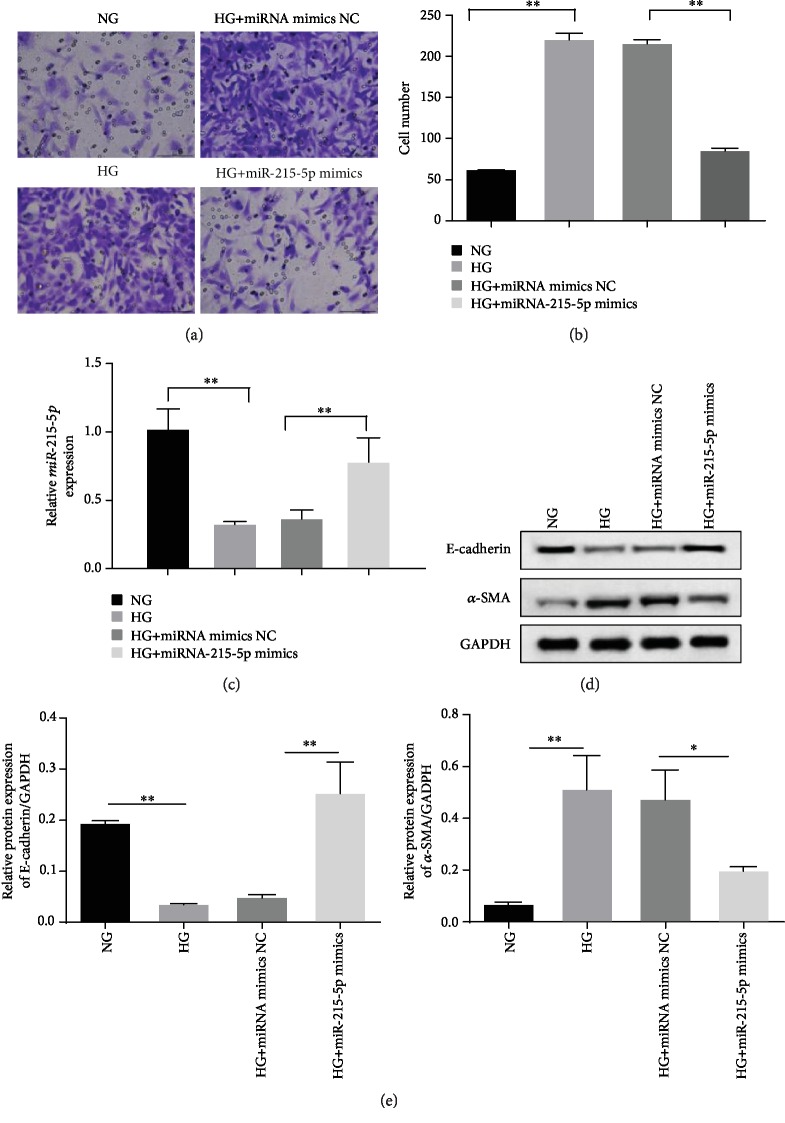
Effects of miR-215-5p on HG-mediated migration of MPC5 cells. (a) Cell migration was assayed by Transwell assay in HG-induced MPC5 cells treated with miR-215-5p mimic. (b) The number of migratory cells was analyzed by ImageJ software. (c) The expression of miR-215-5p was measured by qRT-PCR in HG-induced MPC5 cells treated with miR-215-5p mimic. (d) The expression of E-cadherin and a-SMA was measured by western blotting under miR-215-5p mimic treatment. (e) Grayscale quantitation of E-cadherin and *α*-SMA protein expression. ^∗^*P* < 0.05; ^∗∗^*P* < 0.01.

It has been demonstrated that miRNA-215 was a negative regulator for cell migration and invasion in many diseases [23–25]. When the expression of miRNA-215-5p using mimics in MPC5 cells was forced, its decline was recovered in the presence of HG (Figure 3(a)). Under the same circumstances, we discovered that miRNA-215-5p mimics strikingly exhibited strong inhibition effects on HG-induced MPC5 cell migration ability (Figures 3(b) and 3(c)). Besides, western blotting results demonstrated that high glucose powerfully inhibited the expression of E-cadherin (epithelial cell marker) and promoted the accumulation of *α*-SMA (mesenchymal cell marker) compared to normal control (NG groups), but miRNA-215-5p mimic transfection observably reversed HG-evoked alterations of E-cadherin and *α*-SMA (Figure 3(d)). Quantitative analysis of the grayscale images of the bands further confirmed that miRNA-215-5p mimics restored the HG-aroused EMT progress of podocytes (Figure 3(e)).

### 3.4. ADSCs-Exos Mediate the Shuttling of miR-215-5p to Podocyte

**Figure 4:**
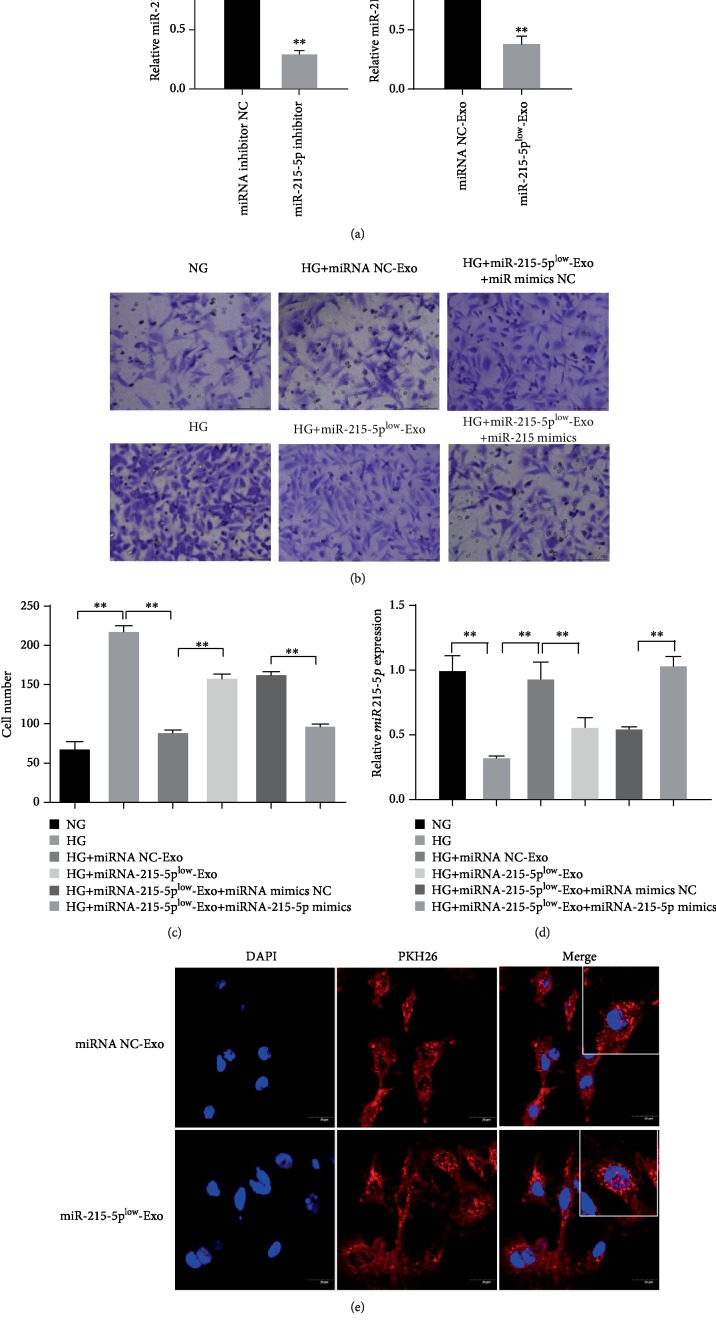
Effects of miR-215-5p loaded in ADSCs-Exos on HG-mediated migration of MPC5 cells. (a) The expression of miR-215-5p was measured by qRT-PCR in ADSC treated with miR-215-5p inhibitor. (b) The level of miR-215-5p was analyzed by qRT-PCR in exosomes from ADSCs treated with miR-215-5p inhibitor. (c) Cell migration was evaluated by Transwell assay in HG-induced MPC5 cells treated with exosomes with low miR-215-5p expression and miR-215-5p mimic. (d) The number of migratory cells was analyzed by ImageJ software, and the expression of miR-215-5p was measured by qRT-PCR. (e) Tracking of the PKH26-labeled ADSC-Exos with low miR-215-5p expression or normal miR-215-5p expression in HG-induced MPC5 cells. ^∗^*P* < 0.05; ^∗∗^*P* < 0.01.

Since miRNA-215-5p had a negative effect on cell migration, we speculated whether the ADSCs-Exo-mediated migration inhibition was related to the ADSCs-Exo-launched increase of miRNA-215-5p in the presence of HG. To solve this query, we first knocked down the expression of miR-215-5p in ADSCs by using the miR-215-5p inhibitor. As shown in Figure 4(a), miR-215-5p inhibitor significantly decreased the expression of miR-215-5p in ADSC cells compared with noninhibitors (Figure 4(a)). Meanwhile, exosomes secreted from ADSCs transfected with the miR-215-5p inhibitor showed a low level of miR-215-5p in comparison with exosomes derived from ADSCs transfected with the negative control inhibitor, which suggested that inhibition of miR-215-5p in ADSCs thereby caused the downregulation of miR-215-5p in exosomes extracted from these cells (Figure 4(b)). Next, with the Transwell assay, we revealed that ADSCs-Exos with low expression of miR-215-5p (miR-215-5p^low^ Exo) exhibited weaker inhibition effects on HG-induced podocyte migration than normal ADSCs-Exos. However, transfection with miR-215-5p mimics in MPC5 cells reversed the effect (Figures 4(c) and 4(d)). Meanwhile, the miR-215-5p level was low in MPC5 cells treated with miR-215-5p^low^ Exo compared to cells exposed to normal ADSCs-Exo, whereas miR-215-5p mimics transfection made up for the lack of miR-215-5p induced by miR-215-5p^low^ Exo (Figure 4(e)). Moreover, after staining with PKH26 (to mark the membranes of exosomes), exosomes from normal ADSCs and ADSCs with low miR-215-5p were cocultured with MPC5 cells and the fluorescence was tracked by IF assay. The results showed that both miR-215-5p^low^ Exo and mi-RNA NC-Exo were internalized into MPC5 cells and did not present any differences between them, suggesting ADSCs-Exo with depressed miR-215-5p did not affect exosome uptake by podocytes (Figure 4(f)). These results indicate that miR-215-5p packaged in ADSCs-Exos was the determining factor for the repair behavior mediated by ADSCs-Exos in MPC5 cells.

### 3.5. ZEB2 Is the Target Gene of miR-215-5p

**Figure 5:**
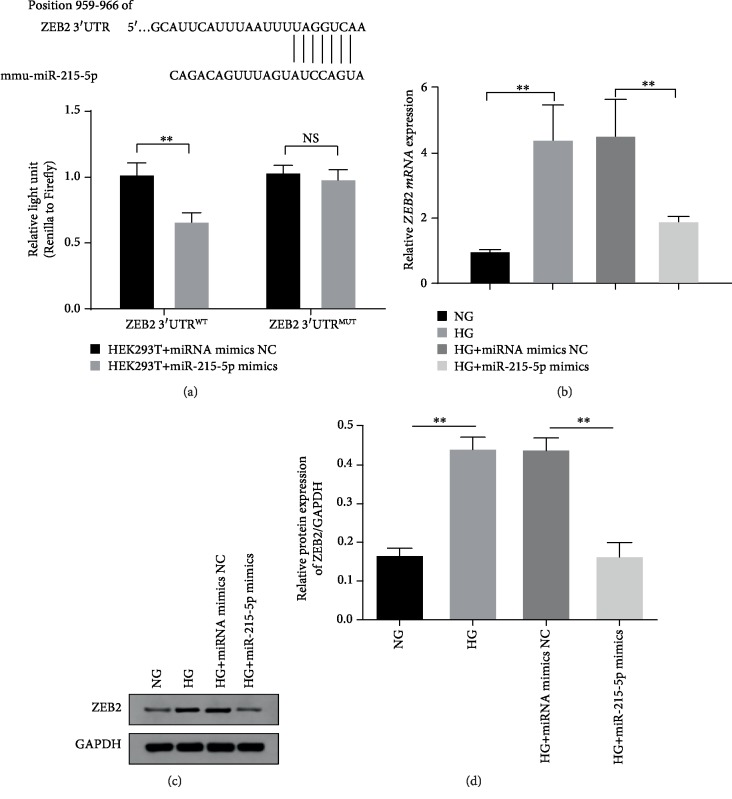
The correlation between miR-215-5p and ZEB2 in podocyte migration. (a) Targeted binding sites between miR-215-5p and *ZEB2*-3′-UTR sequences were predicted by TargetScan database and effect of miR-215-5p on transcription activity of *ZEB2* determined by luciferase report assay in 293 cells. (b) The expression of *ZEB2* was measured by qRT-PCR in 293 cells treated with miR-215-5p mimic. (c) The protein level of ZEB2 was measured by western blotting in MPC5 cells treated with miR-215-5p mimic. (d) Grayscale quantitation of ZEB2 protein expression. ^∗^*P* < 0.05; ^∗∗^*P* < 0.01.

Zinc finger E-box-binding homeobox 2 (ZEB2), known as a DNA-binding transcription factor, is involved in the process of EMT, migration, and invasion [26]. Combined with TargetScan online database, we found that there were potential binding sites between miR-215-5p and ZEB2 (Figure 5(a)). And miR-215-5p mimics significantly blunted the transcriptional activity of wild-type *ZEB2* but failed to impact the mutation-type *ZEB2* (Figure 5(a)). In MPC5 cells, high glucose notably promoted the transcriptional expression of *ZEB2* and exogenous miR-215-5p effectively blocked HG-induced *ZEB2* accumulation (Figure 5(b)). Additionally, the protein level of ZEB2 was significantly enhanced under the stimulation of HG compared with the NG groups. But overexpression of miR-215-5p vividly inhibited the enhancement of ZEB2 (Figure 5(c)). Quantitative analysis of grayscale images of the bands further confirmed the relationship between ZEB2 and miR-215-5p in podocytes (Figure 5(d)). These results suggest that miR-215-5p inhibits the expression of ZEB2 by directly targeting its 3′-UTR, implying that miR-215-5p negatively regulates ZEB2 activity.

## 4. Discussion

Podocytes cover the outside of glomerular basement membrane and act as the final protective barrier of the kidney and are essential to maintain glomerular filtration function [27]. The disorder of glomerular filtration function leads to proteinuria in patients with diabetes and DN process acceleration [28]. Increasing evidence suggests that podocyte EMT can be induced by high glucose, which is closely associated with podocyte-specific apoptosis and depletion that involves in the onset of proteinuria and the progression of DN [29, 30]. In the present study, ADSCs-Exo preparations significantly reversed the effects of serum from DN patients, and high concentration of HG-triggered podocyte EMT, which provides theoretical support for clinical treatment of DN through regulating EMT progression of podocytes.

Exosomes are the nanosized membrane-bound delivery vesicles, which play a critical role in regulating cellular biofunction by mediating cell-to-cell communication [31]. Exosomes extracted from HG-treated glomerular endothelial cells (GECs) can be internalized by podocytes, leading to podocyte EMT and renal fibrosis [32, 33]. In contrast, some exosomes exert protective effects to be used for treatment of many diseases including podocyte injury and kidney disease. Human umbilical cord mesenchymal stem cell-derived exosomes (hucMSC-Exos) alleviate cisplatin-induced renal oxidative stress injury and apoptosis in rats [34]. Moreover, human urine stem cell-derived exosomes (USCs-Exos) protect podocytes from high-glucose-induced injury, resulting in the inhibition of kidney complications from type I diabetes in rats [11]. Mesenchymal stem cell- (MSC-) derived exosomes prevent the kidney damage caused by hyperglycemia through enhancing autophagy activity and are regarded as a potential new therapeutic strategy for chronic renal injury [35]. In our research, we confirmed that ADSCs-Exo administration repaired podocyte EMT and the damage induced by a high glucose medium *in vitro* but failed to prove the protective role of ADSCs-Exo on podocyte EMT *in vivo.* Whether the protective effect of ADSCs-Exo on podocyte EMT involves in autophagy flux needs further study.

Along with the activation of the EMT process, podocytes cultured in HG medium also show a large number of abnormal microRNA profiles [36]. Exosomes from human cord blood endothelial colony-forming cells partially restored kidney damage caused by ischemic-reperfusion in FBV mice by sending miR-486-5p to inhibit PTEN and repress cell apoptosis [37]. HG addition is able to induce the increase of micro-RNA-346 which is a concentration-dependent effect, and miR-346 mimic significantly inhibits EMT of podocytes by indirectly decreasing the expression of GSK-3*β* [38]. In podocytes, miR-30a significantly enhanced the expression of E-cadherin, an EMT marker, blocking the podocyte EMT by targeting NFATc3 [39]. These studies indicate that miRNAs play a pivotal role in regulating podocyte EMT. There is evidence that exosomes derived from stem cells transport exogenous miRNAs to recipient cells, suggesting potential therapeutic application [40, 41]. Exosomes derived from MSCs overexpressing with miRNA-let7c effectively attenuates renal fibrosis caused by unilateral ureteral obstruction [42]. microRNA-16-5p packaged in USCs-Exo showed powerfully protective effects on diabetic nephropathy in diabetic rats by ameliorating podocyte apoptosis [20]. It has been reported that the level of miR-215 was decreased in diabetic kidney and correlated negatively with *E-cadherin* mRNA (a negative regulator of EMT) in TGF-*β*-induced tubular cells, suggesting decreased miR-215 involved in the DN progress and EMT of proximal tubular cells (NRK52E) [43]. But TGF-*β*1 increased the expression of miR-215, thereby inducing *α*-SMA and EMT progression in mouse mesangial cells (MMCs), suggesting the dual role of miR-215 on EMT regulation in different cells [44]. In our study, we detected the protective effects of miR-215-5p on podocyte EMT and further confirmed that ADSCs-Exo treatment for HG-induced EMT is particularly dependent on the level of miR-215-5p. Here, diverse effects of miR-215-5p on fibrosis were verified in podocytes. However, the effect of miR-215-5p derived from ADSCs on DN *in vivo* needs further investigation.

It has been reported that zinc finger E-box-binding homeobox-2 (ZEB2) was overexpressed in most aggressive cancers, such as bladder cancer, gastric cancer, colorectal cancer, and small cell lung cancer and essential for tumor invasion and EMT process [45–48]. ZEB2 depletion significantly hinders the gene transcription that is involved in cell adhesion and migration and thus impair proliferation and EMT, both in human and in mouse acute myeloid leukemia cells [49]. It has been demonstrated that ZEB2 interacts with the transcription factor Sp1 to activate the expression of mesenchymal genes and vimentin expression, leading to EMT in human cancer cells [47]. ZEB2 can directly bind to conserved E2 boxes of the E-cadherin promoter to repress E-cadherin expression, accelerating EMT and invasion in malignant epithelial tumors [50]. ZEB2 is also involved in the development of some forms of kidney diseases. It has been demonstrated that ZEB2 expression promotes epithelial-to-mesenchymal transition (EMT) in renal cell carcinoma (RCC) and accumulated ZEB2 is an effective biomarker for the poor prognosis of patients with RCC [51]. Additionally, mice with a ZEB2-specific deletion in metanephric mesenchyme led to primary glomerulocystic disease without tubular dilatation, which was absent in control mice, suggesting ZEB2 is required for proximal tubule development and normal glomerulotubular junction formation [52]. microRNAs have been found to be a regulator of ZEB2 and function as the EMT suppressor in many types of cells. Forced expression of microRNA-187 inhibits EMT progression in osteosarcoma by directly targeting the 3′UTR of *ZEB2* [53]. miR-200b overexpression alleviates cell migration and EMT in glioma U251 and U87 cells by negatively regulating ZEB2 expression [54]. Spectacularly, miR-215 inhibits ZEB2 expression by directly targeting effects on ZEB2 and pancreatic cancer cell proliferation, invasion, and migration [55]. miR-215, as a tumor suppressor, suppresses cell migration and apoptosis in human non-small-cell lung cancer by inhibiting ZEB2 [56]. In the present study, miR-215b attenuated the promotional role of HG on ZEB2 expression in podocytes by directly binding to the 3′-UTR of *ZEB2*. However, there was no direct evidence that the shuttling of miR-215 from ADSCs-Exos to podocytes mediates the expression of ZEB2. Also, whether forced expression of ZEB2 may reverse the protection role of ADSCs-Exos on the EMT process of podocytes remains unknown. Thus, further studies are needed to explore more details of the role of ADSCs-Exo/miRNA-215/ZEB2 signaling axis on ADSCs-Exo-mediated amelioration of EMT.

## 5. Conclusion

In summary, ADSC-Exo could alleviate the process of EMT and pathological changes in HG-induced podocytes by delivering functionally active miR-215-5p to podocytes. In the podocyte, ZEB2 was the downstream target of miR-215 and a key regulator of EMT, implying exosomal miR-215-5p might relieve podocyte injury and EMT by regulating ZEB2 expression. Potentially, the ADSC-Exo or a targeted miR-215 approach can serve as a protective strategy towards podocyte EMT.

## Figures and Tables

**Table 1:** Primer sequences in this study.

Gene	Type	Sequence
GAPDH	Forward primer:	5′-ATGGGTGTGAACCACGAGA-3′
	Reverse primer:	5′-CAGGGATGATGTTCTGGGCA-3′

ZEB2	Forward primer:	5′-AGTGGCAGCAGTCCCTTTAT-3′
	Reverse primer:	5′-TCCGTCTTGCAGTCCATCTT-3′

U6	Forward primer:	5′-CGCTTCGGCAGCACATATAC-3′
	Reverse primer:	5′-AAATATGGAACGCTTCACGA-3′

miR-215-5p	Loop primer:	5′-GTCGTATCCAGTGCAGGGTCCGAGGT ATTCGCACTGGATACGACGTCTGTCA-3′
	Forward primer:	5′-TGCGCATGACCTATGATTTGA-3′
	Reverse primer:	5′-CCAGTGCAGGGTCCGAGGTATT-3′

miR-7a-5p	Loop primer	5′-GTCGTATCCAGTGCAGGGTCCGAGGTA TTCGCACTGGATACGACACAACAAAA-3′
	Forward primer:	5′-TGCGCTGGAAGACTAGTGATTT-3′
	Reverse primer:	5′-CCAGTGCAGGGTCCGAGGTATT-3′

miR-879-5p	Loop primer:	5′-GTCGTATCCAGTGCAGGGTCCGAGGTA TTCGCACTGGATACGACGGCTTAGA-3′
	Forward primer:	5′-TGCGCAGAGGCTTATAGCTCT-3′

miR-217-5p	Loop primer:	5′-GTCGTATCCAGTGCAGGGTCCGAGGTA TTCGCACTGGATACGACTCCAGTCA-3′
	Forward primer:	5′-TGCGCTACTGCATCAGGAACTGA-3′
	Reverse primer:	5′-CCAGTGCAGGGTCCGAGGTATT-3′

miR-3066-5p	Loop primer	5′-GTCGTATCCAGTGCAGGGTCCGAGGTA TTCGCACTGGATACGACCTACTTAA-3′
	Forward primer:	5′-TGCGCTTGGTTGCTGTAGATTA-3′
	Reverse primer:	5′-CCAGTGCAGGGTCCGAGGTATT-3′

## Data Availability

The [DATA TYPE] data used to support the findings of this study are included within the article.

## Supplementary Materials

Supplementary Materials Figure S1: identification of exosomes derived from mouse ADSCs. (A) Representative micrographs of transmission electron microscopy of purified exosomes derived from ADSCs (ADSCs-Exo). Scale bar = 100 nm. ADSCs were harvested from 8-week-old C57BL/KsJ db/m male mice by utilizing the collagenase digestion method. (B) CD9, CD63, and CD81 expressions as determined by western blotting in exosomes derived from ADSCs. (C) Purity identification of ADSCs-Exo by flow cytometry using CD9, CD63, and CD81 antibodies. Figure S2: evaluation of the inhibitory effect of EMT-related miRNAs. MPC5 cells were first transfected with miRNA inhibitor negative control, miR-215-5p inhibitor, miR-879-5p inhibitor, miRNA-217a-5p inhibitor, miRNA-3066-5p inhibitor, and miRNA-7a-5p inhibitor for 48 h. Then, the expression of miR-215-5p (A), miR-879-5p (B), miRNA-217a-5p (C), miRNA-3066-5p (D), and miRNA-7a-5p (E) was determined by qRT-PCR in MPC5 cells. ^∗^*P* < 0.05; ^∗∗^*P* < 0.01.
