# Supplementary material for: Exosomal miRNA-215-5p Derived from Adipose-Derived Stem Cells Attenuates Epithelial–Mesenchymal Transition of Podocytes by Inhibiting ZEB2
Source: Biomed Res Int. 2020 Feb 21;2020:2685305. doi: 10.1155/2020/2685305 (PMC7057016; doi:10.1155/2020/2685305)
Supplement: Supplementary Materials — Figure S1: identification of exosomes derived from mouse ADSCs. (A) Representative micrographs of transmission electron microscopy of purified exosomes derived from ADSCs (ADSCs-Exo). Scale bar = 100 nm. ADSCs were harvested from 8-week-old C57BL/KsJ db/m male mice by utilizing the collagenase digestion method. (B) CD9, CD63, and CD81 expressions as determined by western blotting in exosomes derived from ADSCs. (C) Purity identification of ADSCs-Exo by flow cytometry using CD9, CD63, and CD81 antibodies. Figure S2: evaluation of the inhibitory effect of EMT-related miRNAs. MPC5 cells were first transfected with miRNA inhibitor negative control, miR-215-5p inhibitor, miR-879-5p inhibitor, miRNA-217a-5p inhibitor, miRNA-3066-5p inhibitor, and miRNA-7a-5p inhibitor for 48 h. Then, the expression of miR-215-5p (A), miR-879-5p (B), miRNA-217a-5p (C), miRNA-3066-5p (D), and miRNA-7a-5p (E) was determined by qRT-PCR in MPC5 cells. ∗P < 0.05; ∗∗P < 0.01. [file 2685305.f1.pdf]

Figure. S1

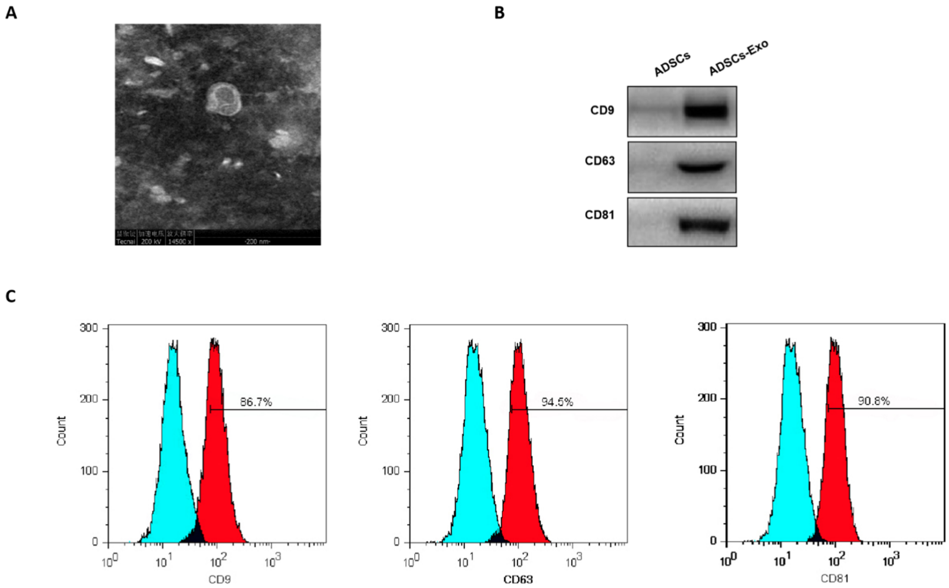

Figure S1. Identification of exosomes derived from mouse ADSCs. (A) Representative micrographs of transmission electron microscopy of purified exosomes derived from ADSCs, Scale bar = 100 nm. **(B)** Identification of exosomes derived from ADSCs, CD9, CD63, and CD81 expression by Western blotting. **(C)** Purity identification of ADSCs-Exo by flow cytometry using CD9, CD63 and CD81 antibodies.

Figure. S2

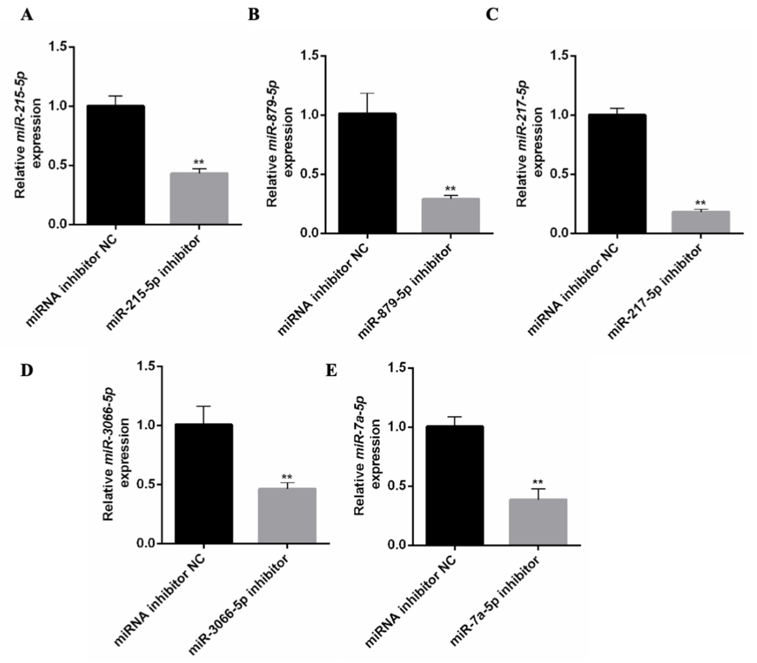

Figure S2. Evaluation of the inhibitory effect of EMT-related miRNAs. The expression of miR-215-5p **(A)**, miR-879-5p**(B)**, miRNA-217a-5p**(C)**, miRNA-3066-5p**(D)** and miRNA-7a-5p**(E)** were determined by qRT-PCR in MPC5 cells. *, P <0.05; **, P <0.01.
